# Real-World Evidence Prediction of a Phase IV Oncology Trial: Comparative Degarelix vs Leuprolide Safety

**DOI:** 10.1093/jncics/pkac049

**Published:** 2022-08-10

**Authors:** David Merola, Sebastian Schneeweiss, Sushama K Sreedhara, Luke E Zabotka, Kenneth Quinto, John Concato, Shirley V Wang

**Affiliations:** Division of Pharmacoepidemiology and Pharmacoeconomics, Department of Medicine, Brigham and Women’s Hospital, Harvard Medical School, Boston, MA, USA; Department of Epidemiology, Harvard T.H. Chan School of Public Health, Boston, MA, USA; Division of Pharmacoepidemiology and Pharmacoeconomics, Department of Medicine, Brigham and Women’s Hospital, Harvard Medical School, Boston, MA, USA; Department of Epidemiology, Harvard T.H. Chan School of Public Health, Boston, MA, USA; Division of Pharmacoepidemiology and Pharmacoeconomics, Department of Medicine, Brigham and Women’s Hospital, Harvard Medical School, Boston, MA, USA; Division of Pharmacoepidemiology and Pharmacoeconomics, Department of Medicine, Brigham and Women’s Hospital, Harvard Medical School, Boston, MA, USA; Center for Drug Evaluation and Research, Food and Drug Administration, Silver Spring, MD, USA; Center for Drug Evaluation and Research, Food and Drug Administration, Silver Spring, MD, USA; Division of Pharmacoepidemiology and Pharmacoeconomics, Department of Medicine, Brigham and Women’s Hospital, Harvard Medical School, Boston, MA, USA

## Abstract

**Background:**

Medical and regulatory communities are increasingly interested in the utility of real-world evidence (RWE) for answering questions pertaining to drug safety and effectiveness, but concerns about validity remain. A principled approach to conducting RWE studies may alleviate concerns and increase confidence in findings. This study sought to predict the findings from the PRONOUNCE trial using a principled approach to generating RWE.

**Methods:**

This propensity score–matched observational cohort study used 3 claims databases to compare the occurrence of major adverse cardiovascular events among initiators of degarelix vs leuprolide. Patients were included if they had a history of prostate cancer and atherosclerotic cardiovascular disease. Patients were excluded if they did not have continuous database enrollment in the year before treatment initiation, were exposed to androgen deprivation therapy or experienced an acute cardiovascular event within 30 days before treatment initiation, or had a history or risk factors of QT prolongation.

**Results:**

There were 12 448 leuprolide and 1969 degarelix study-eligible patients before matching, with 1887 in each arm after propensity score matching. The results for major adverse cardiovascular events comparing degarelix with leuprolide in the observational analysis (hazard ratio = 1.35, 95% confidence interval = 0.94 to 1.93) was consistent with the subsequently released PRONOUNCE result (hazard ratio = 1.28, 95% confidence interval  = 0.59 to 2.79).

**Conclusions:**

This study successfully predicted the result of a comparative cardiovascular safety trial in the oncology setting. Although the findings are encouraging, limitations of measuring cancer stage and tumor progression are representative of challenges in attempting to generalize whether claims-based RWE can be used as actionable evidence.

Medical and regulatory communities in the United States have become increasingly interested in real-world data and real-world evidence (RWE) to answer drug safety and effectiveness questions. RWE studies are conducted using real-world data, defined as data collected during routine clinical care, such as administrative claims, or data derived from mobile devices and disease registries (1). Administrative claims contain longitudinal health-related information on prescription drug use and medical diagnoses and procedures. These data have been used for a variety of purposes by various health-care stakeholders, including evaluation of labeled indications in routine clinical care (2), evaluation of potential for off-label indications (3), evaluation of adverse reactions and overall safety profile (4), and to identify “external” control groups representing alternative treatments or standard of care (5,6).

Despite many potential applications of data collected from routine health-care delivery, the credibility of RWE remains controversial. Poor data quality, inappropriate study choices, confounding, and bias pose potential threats to validity of findings based on RWE studies (7). These challenges highlight the need for a principled approach to analysis of longitudinal health-care databases as well as a framework for understanding the manners in which RWE can be effectively applied.

To better understand the types of questions that can be answered with health-care databases, our team has attempted to emulate or predict the results of 30+ randomized trials through a formalized process (8-10). This approach involves a series of prespecified checkpoints when developing the protocol, preregistration of the protocol, and a thorough assessment of emulation differences and bias after implementation. Such a principled approach to conducting RWE may help alleviate concerns and increase confidence in findings.

To date, few oncologic clinical trial emulations have been completed (11). The current research emulates a randomized comparative safety study, the PRONOUNCE trial (12,13), which was designed to evaluate the comparative risk of major adverse cardiovascular events (MACE), defined as a composite of all-cause mortality, nonfatal myocardial infarction, or nonfatal stroke, among advanced prostate cancer patients treated with androgen deprivation therapy. The trial was intended to address conflicting reports of increased cardiovascular risk among patients treated with gonadotropin-releasing hormone agonists vs antagonists (14-19). Using a previously described approach to clinical trial emulation, we aimed to predict the findings of the PRONOUNCE trial before trial results were published (10).

## Methods

### Data Sources

Three administrative claims databases were used in this study: Optum Clinformatics (December 24, 2008-June 30, 2020), IBM MarketScan (December 24, 2008-December 31, 2018), and a subset of Medicare claims data consisting of diabetes patients only (December 24, 2008-December 31, 2017). Although the Medicare data cut was not a random sample of Medicare beneficiaries, the diabetic patient sample is enriched with high cardiovascular comorbidity, a key inclusion criterion for the trial. These longitudinal, patient-level databases contain diagnostic and procedural information in the form of International Classification of Diseases (ICD), Current Procedural Terminology, and Healthcare Common Procedure Coding System codes submitted in both inpatient and outpatient health-care claims. Additionally, outpatient prescription claims are recorded. These clinical codes were used to construct algorithms for inclusion-exclusion criteria, exposure, outcome, and baseline covariates—key parameters necessary to create a study population and design congruent with the PRONOUNCE trial participants and design.

### Study Population

**Figure 1.**
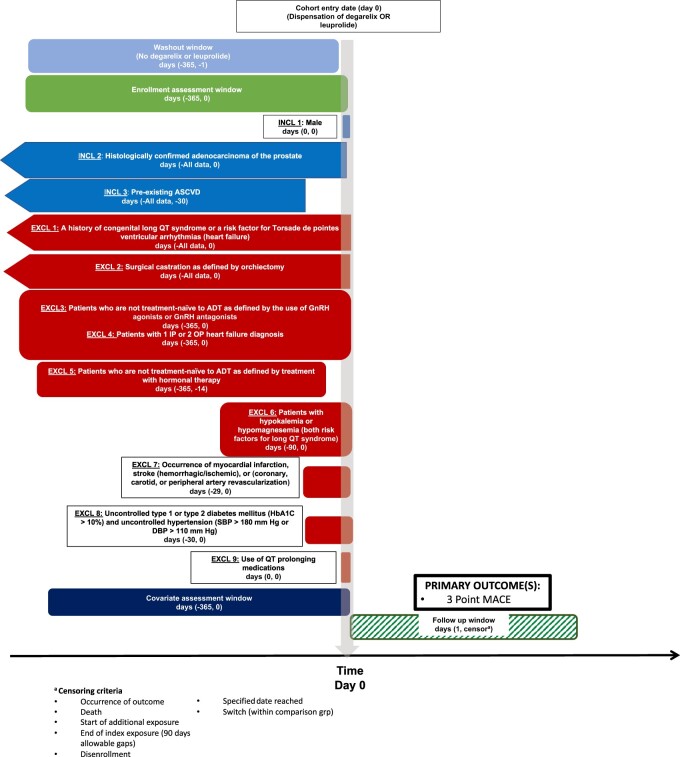
A schematic of the study design. ADT = androgen deprivation therapy; ASCVD = atherosclerotic cardiovascular disease; DBP = diastolic blood pressure; EXCL = exclusion criterion; GnRH = gonadotropin releasing hormone; HbA1C = hemoglobin A1C; INCL = inclusion criterion; IP = inpatient; MACE = major adverse cardiovascular event; OP = outpatient; SBP = systolic blood pressure.

We conducted an active-comparator, new-user, cohort study comparing patients who initiated degarelix vs leuprolide emulating the PRONOUNCE trial design (Figure 1) (20). This nonrandomized emulation approach has been shown to reduce bias from confounding, differential surveillance, and immortal time (21).

Eligibility criteria were adapted to mimic the PRONOUNCE trial. Analogous to the date of random assignment in the trial, the index date was the initiation date of either degarelix or leuprolide after at least a 365-day washout; the cohort entry date was selected after applying all selection criteria. Included patients had at least 1 diagnosis code indicating prostate cancer, were male, and had a history of atherosclerotic cardiovascular disease on or before the index date. Patients were excluded if they were not treatment-naïve with respect to androgen deprivation therapy; had a record indicating an ICD code(s) for uncontrolled diabetes or hypertension within 30 days before treatment initiation; had a history of long QT syndrome or risk factors thereof (ie, heart failure, hypokalemia, or medications known to prolong the QT interval); or had an acute myocardial infarction, stroke, or revascularization procedure within 30 days before treatment initiation. Additionally, all patients were required to have continuous enrollment for 365 days before cohort entry to ensure incident use of the study drugs and adequate capture of confounders at baseline.

Several selection criteria applied in the PRONOUNCE trial could not be applied to the observational cohort due to poor capture in claims data. Specifically, patients in the observational cohort were not required to have established tumor staging information, angiography-verified stenosis/occlusion of vessels, or plans for cardiac surgery at the time of treatment initiation. Detailed information on how eligibility criteria from the PRONOUNCE trial were adapted in our RWE study can be seen on clinicaltrials.gov **(**NCT04897958).

### Exposure Definition

Study exposures were defined by the presence of Healthcare Common Procedure Coding System/Current Procedural Terminology codes (degarelix: J9155; leuprolide: J1950, C9430, J9217-J9219) or outpatient prescription drug claims indicating degarelix dispensation. Incident use of these drugs was defined by having no records of degarelix, leuprolide, or other androgen deprivation therapy in the previous 365 days.

### Outcome Definition

The primary outcome, MACE, was a composite of death or inpatient ICD diagnosis codes for nonfatal myocardial infarction (any diagnosis position) and stroke (primary diagnosis position). Billing codes used to define MACE and details on how death was defined in each database can be found on clinicaltrials.gov (NCT04897958). Secondary outcomes were the occurrence of individual components of the MACE outcome (ie, nonfatal myocardial infarction, nonfatal stroke, or all-cause mortality).

### Feasibility Analysis

Following a predefined process (10), we conducted an initial feasibility analysis to evaluate event counts in our data sources to estimate a treatment effect with the same power as the PRONOUNCE trial. This process included feasibility counts (unstratified by treatment) and power calculations, before and after matching on a propensity score, and evaluation of balance diagnostics such as pre- and postmatching c-statistics and standardized differences for baseline covariates. The c-statistic before matching provides a sense of how divergent the compared treatment arms are in terms of measured characteristics. If balance is achieved, the postmatching c-statistic is expected to be near 0.5 (22). Similarly, a rule of thumb often used to define meaningful imbalance on a covariate is a standardized difference of greater than 0.1 (22).

After estimating outcome rates unstratified by exposure status, we applied similar assumptions as used for the trial’s power calculation; namely, a 2-sided alpha level of .05 and an occurrence of 94 MACE events among a matched cohort of 3774 patients. We calculated 93% power to detect a hazard ratio for degarelix vs leuprolide of 0.49 after pooling estimates across 3 databases.

### Confounding Factors and Balance Diagnostics

To control for confounding, over 100 potential predictors of MACE were included in the propensity score model. Each of these covariates were measured in the 365 days before (and including) the index date. The covariates included in the propensity score model were related to demographics, chronic disease medication, cardiovascular events, general health and mortality, and prostate cancer progression. We also adjusted for markers of health-care use to mitigate the potential for healthy user bias (23). To account for a potential differential risk of death between treatment groups at baseline, a published prostate cancer comorbidity index (PCCI) was used (24). This PCCI is a validated claims-based algorithm designed to predict mortality among men with prostate cancer and includes 24 categories indicating organ dysfunction and chronic disease (eg, dementia, cardiovascular disease, nonprostate malignancies, renal disease, etc). The index can serve as a proxy for Gleason score, which is not available in claims (24). Balance in these confounders was assessed with the postmatching c-statistic and standardized difference, with values of 0.5 and less than 0.1 indicating balance between treatment groups, respectively (22).

### Statistical Analysis

The Aetion Evidence Platform (25,26) was used to generate all study variables and conduct statistical analyses. The platform provides an audit trail to facilitate transparency of what analyses were conducted and when.

Within all 3 databases, a Cox proportional hazards model was used to estimate the hazard ratio for MACE among users of degarelix vs leuprolide in a 1:1 propensity score–matched population. Proportionality of the hazards was verified graphically with Schoenfeld residuals plots. Matching was conducted by first discarding patients with propensity scores in the upper and lower 2.5th percentile of the combined propensity score distribution in the degarelix and leuprolide recipients. Then, each patient initiating degarelix was paired to a patient initiating leuprolide using a 1% caliper. Although the PRONOUNCE trial focused on an intention-to-treat analysis, to mimic the high adherence observed in trials, our primary analysis was an on-treatment analysis where patients were censored if they discontinued or switched therapy (27). An on-treatment analysis reduces the potential for bias from misclassifying exposure time that would be found in an intention-to-treat analysis if, as is often observed in clinical practice, there is a high rate of treatment discontinuation. Follow-up began on the day after cohort entry and proceed until the earliest of the following: 1) treatment discontinuation, defined by a 90-day gap between treatment records or crossover in treatment; 2) database disenrollment; 3) administrative end of data; 4) 365 days of follow-up; or 5) experience of study outcome event. Separate estimates from each database were pooled together using a fixed-effects meta-analysis. Tests of statistical significance were 2-sided with an alpha level of .05.

### Sensitivity Analyses

To assess the robustness of our results to our assumptions regarding continuous treatment, we conducted an intention-to-treat (ITT) analysis that did not censor for discontinuation or switch of treatment and was otherwise carried out in the same manner as the primary on-treatment analysis. Additionally, we assessed the sensitivity of our results to the death component of the MACE outcome by repeating the primary analysis after removal of death as a component of MACE (ie, MACE was redefined as nonfatal myocardial infarction or stroke only). This analysis was done for 2 reasons: 1) mortality is partially captured in the MarketScan database through (inpatient only) discharge status codes, whereas mortality is more completely captured across multiple sources in the Medicare and Clinformatics databases; and 2) although we had good proxy measures to facilitate adjustment for potential confounding from imbalances in risk factors for cardiovascular events and cardiovascular-related death, our capture of risk factors for cancer-related death at baseline was limited.

### Preregistration of Protocol Before Ongoing Trial Results Are Publicized

After passing the prespecified check points and finalizing our analytic plan, we registered our study protocol on clinicaltrials.gov on May 24, 2021 (NCT04897958), and, according to our software’s audit trail, our primary study results were first available on the same day. The PRONOUNCE trial results were made public on August 30, 2021 (12).

## Results

After applying eligibility criteria, the unmatched study population included 12 448 leuprolide and 1969 degarelix initiators. In the matched population, there were 546, 415, and 926 patients in each treatment arm within the Clinformatics, MarketScan, and Medicare databases, respectively. Relative to leuprolide initiators, degarelix initiators tended to exhibit greater health-care use with respect to imaging and diagnostics used in prostate cancer and had more pneumonia vaccination (Table 1). Patients in the MarketScan data tended to be younger with fewer comorbidities (eg, acute or old myocardial infarction events, angina, diabetes with complications, smoking, PCCI) relative to the other databases. Patients initiating degarelix also tended to have a greater mean PCCI. These differences, however, were minimal in the matched cohort, with all standardized differences less than 0.1 (Table 1), and c-statistics in all 3 databases moving from 0.7 to 0.6 after matching (see clinicaltrials.gov NCT04897958). Residual imbalances in the matched c-statistic may be due to the large number of covariates relative to the number of matched pairs.

**Table 1.** Baseline demographics and clinical characteristics of pooled study cohort^a^

Variable	Unmatched			Matched		
	Leuprolide	Degarelix	St. Diff	Leuprolide	Degarelix	St. Diff
No. of patients	12 448	1969		1887	1887	
Mean age (SD), y	75.93 (7.48)	76.10 (7.49)	−0.02	76.24 (6.74)	76.18 (6.87)	0.01
Region, No. (%)						
Northeast	1807 (14.5)	388 (19.7)	−0.14	374 (19.8)	366 (19.4)	0.01
North central	3695 (29.7)	518 (26.3)	0.08	484 (25.6)	498 (26.4)	−0.02
South	4602 (37.0)	733 (37.2)	0.00	721 (38.2)	703 (37.3)	0.02
West	2333 (18.7)	327 (16.6)	0.06	308 (16.3)	320 (17.0)	−0.02
Race^b^, No. (%)						
Asian	160 (1.3)	19 (1.0)	0.03	13 (0.7)	19 (1.0)	−0.03
Black	974 (7.8)	148 (7.5)	0.01	159 (8.4)	146 (7.7)	0.03
Hispanic	363 (2.9)	49 (2.5)	0.02	46 (2.4)	46 (2.4)	0.00
North American Native	28 (0.5)	—	0.01	—	—	−0.02
White	7545 (60.6)	1254 (63.7)	−0.06	1233 (65.3)	1241 (65.8)	−0.01
Unknown	3378 (27.1)	495 (25.1)	0.04	433 (22.9)	431 (22.8)	0.00
Cardiovascular event prognosticators, No. (%)						
Acute or old MI	4180 (33.6)	683 (34.7)	−0.02	658 (34.9)	654 (34.7)	0.00
Anxiety	752 (6.0)	119 (6.0)	0.00	112 (5.9)	112 (5.9)	0.00
Atrial fibrillation	2168 (17.4)	381 (19.3)	−0.05	333 (17.6)	366 (19.4)	−0.05
Coronary atherosclerosis	9687 (77.8)	1567 (79.6)	−0.04	1505 (79.8)	1496 (79.3)	0.01
Revascularization (angioplasty/stent/coronary bypass graft)	1238 (9.9)	156 (7.9)	0.07	133 (7.0)	145 (7.7)	−0.03
Diabetes						
With complications	6296 (50.6)	1007 (51.1)	−0.01	988 (52.4)	968 (51.3)	0.02
Without complications	3895 (31.3)	629 (31.9)	−0.01	587 (31.1)	601 (31.8)	−0.02
DVT	515 (4.1)	76 (3.9)	0.01	76 (4.0)	71 (3.8)	0.01
Edema	1243 (10.0)	236 (12.0)	−0.06	226 (12.0)	223 (11.8)	0.01
Erectile dysfunction	1425 (11.4)	249 (12.6)	−0.04	239 (12.7)	240 (12.7)	0.00
Foot ulcer	404 (3.2)	82 (4.2)	−0.05	69 (3.7)	76 (4.0)	−0.02
Hyperlipidemia	10 236 (82.2)	1671 (84.9)	−0.07	1596 (84.6)	1600 (84.8)	−0.01
Hypertension	10 808 (86.8)	1733 (88.0)	−0.04	1652 (87.5)	1664 (88.2)	−0.02
Intracranial or retroperitoneal hemorrhage	166 (1.3)	27 (1.4)	−0.01	23 (1.2)	25 (1.3)	−0.01
Ischemic heart disease	10 276 (82.6)	1643 (83.4)	−0.02	1581 (83.8)	1567 (83.0)	0.02
Ischemic stroke	2651 (21.3)	430 (21.8)	−0.01	406 (21.5)	406 (21.5)	0.00
Major trauma	723 (5.8)	107 (5.4)	0.02	108 (5.7)	100 (5.3)	0.02
Obesity	1752 (14.1)	279 (14.2)	0.00	268 (14.2)	263 (13.9)	0.01
Other disorders of thyroid gland	443 (3.6)	69 (3.5)	0.01	62 (3.3)	67 (3.6)	−0.02
PE	197 (1.6)	26 (1.3)	0.03	22 (1.2)	24 (1.3)	−0.01
Peripheral vascular disease	3737 (30.0)	599 (30.4)	−0.01	584 (30.9)	568 (30.1)	0.02
Stable angina	1535 (12.3)	235 (11.9)	0.01	207 (11.0)	221 (11.7)	−0.02
Systemic embolism	218 (1.8)	31 (1.6)	0.02	24 (1.3)	27 (1.4)	−0.01
TIA	419 (3.4)	68 (3.5)	−0.01	62 (3.3)	65 (3.4)	−0.01
Prostate cancer prognosticators, No. (%)						
Radiation therapies used in prostate cancer	53 (0.4)	—	0.00	—	—	0.00
Prostatectomy	395 (3.2)	51 (2.6)	0.04	52 (2.8)	49 (2.6)	0.01
PSA test frequency, mean (SD)	2.21 (2.20)	2.10 (1.55)	0.06	2.04 (1.40)	2.10 (1.41)	0.00
Prostate cancer comorbidity index, mean (SD)	35.44 (5.78)	36.38 (6.16)	−0.16	36.33 (5.55)	36.28 (5.35)	0.00

a Percentages may not add to 100% due to rounding. Values less than 11 have been suppressed with an em-dash to preserve patient privacy in accordance with our data use agreements. DVT = deep vein thrombosis; MI = myocardial infarction; PE = pulmonary embolism; PSA = prostate specific antigen; St. Diff = standardized difference; TIA = transient ischemic attack.

b Race information was unavailable in the MarketScan database.

**Figure 2.**
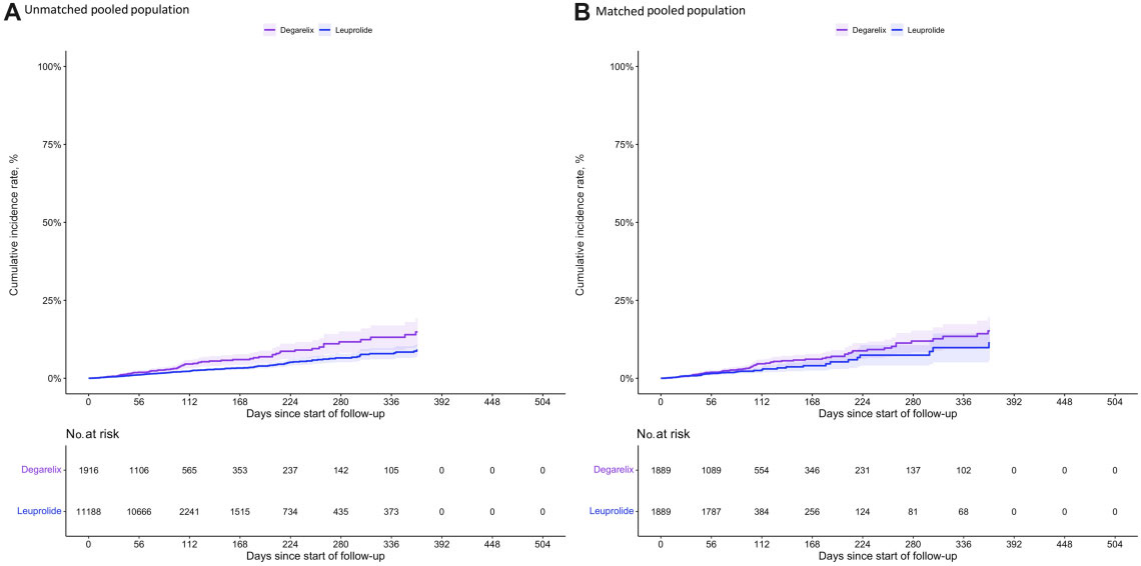
Inverted Kaplan-Meier estimates of cumulative incidence of the major adverse cardiovascular event (MACE). **A**) Cumulative incidence of MACE before matching and after propensity score trimming in the overall (pooled) study population. **B**) Cumulative incidence of MACE after matching in the overall (pooled) study population.

As illustrated in Table 2, our primary analysis produced a similar estimated relative hazard of MACE for degarelix initiators compared with leuprolide initiators to the PRONOUNCE trial (observational hazard ratio [HR] = 1.35, 95% confidence interval [CI] = 0.94 to 1.93; PRONOUNCE HR = 1.28, 95% CI = 0.59 to 2.79). The cumulative MACE incidence over the study period before and after matching for the pooled analysis is shown in Figure 2. Although the MACE rate varied between individual databases, there appeared to be a greater overall incidence of MACE among degarelix initiators over the on-treatment follow-up. Notably, the median follow-up was shorter in the degarelix group (69 days [interquartile range = 32.5-124.5]) compared with the leuprolide group (89 days [interquartile range = 89-89]) in the pooled population. Variability in the absolute rates of MACE between our 3 data sources could be attributed to differences in the underlying patient populations that comprise them. With respect to the primary outcome, the MarketScan and Medicare cohorts produced estimates closer to the null and the Optum Clinformatics cohort produced estimates further from the null (*P*_homogeneity_ = .567) (Supplementary Table 1, available online). MACE components (particularly acute myocardial infarction and stroke) had similar point estimates as the clinical trial (Table 2). Although confidence intervals were wide, the hazard ratio for all-cause mortality (Table 2) had point estimates on the opposite side of null compared with the PRONOUNCE trial (observational HR = 1.41 and 95% CI = 0.90 to 2.21 vs PRONOUNCE HR = 0.84 and 95% CI = 0.32 to 2.18).

**Table 2.** Primary and secondary outcomes^a,b^

Outcome	Real-world data			PRONOUNCE trial		
	Degarelix	Leuprolide	HR (95% CI)^c^	Degarelix	Leuprolide	HR (95% CI)^c^
	(n = 1889)	(n = 1889)		(n = 275)	(n = 269)	
	No. of events (%)	No. of events (%)		No. of events (%)	No. of events (%)	
MACE	73 (3.9)	55 (2.9)	1.35 (0.94 to 1.93)	15 (5.5)	11 (4.1)	1.28 (0.59 to 2.79)
MACE components						
All-cause mortality	50 (2.6)	35 (1.9)	1.41 (0.90 to 2.21)	8 (2.9)	9 (3.3)	0.84 (0.32 to 2.18)
Acute myocardial infarction	25 (1.3)	17 (0.9)	1.52 (0.80 to 2.89)	5 (1.8)	3 (1.1)	1.59 (0.38 to 6.67)
Stroke	7 (0.4)	6 (0.3)	1.05 (0.35 to 3.20)	3 (1.1)	3 (1.1)	0.90 (0.18 to 4.46)
Composite myocardial infarction and stroke	33 (1.7)	29 (1.5)	1.27 (0.76 to 2.12)	—	—	—

a The availability of mortality information varied by database. Medicare and Clinformatics included complete information on all-cause mortality, while MarketScan only included information on in-hospital death. CI = confidence interval; HR = hazard ratio; MACE = major adverse cardiovascular event.

b Median follow-up time to ascertain MACE events in the real-world data study was 89 days (interquartile range = 61-116), which was shorter than that of the PRONOUNCE trial.

c Reference group is leuprolide.

The ITT analysis of the observational study (HR = 1.42, 95% CI = 1.14 to 1.76) were also similar to the randomized trial (HR = 1.32, 95% CI = 0.61 to 2.87). Lastly, repeating our primary analysis after removing the death component from our primary (Supplementary Table 2, available online) endpoint drove the hazard ratio closer to the null in the ITT analysis (HR = 1.03, 95% CI = 0.75 to 1.44), whereas the on-treatment analysis remained largely stable (HR = 1.27, 95% CI = 0.76 to 2.12).

## Discussion

In this observational cohort study emulating the PRONOUNCE trial design of advanced prostate cancer patients with cardiovascular disease history, we predicted results consistent with a non-statistically significant increased MACE risk among degarelix vs leuprolide initiators, involving wide confidence intervals. This result was largely consistent with the PRONOUNCE trial results, which reported a modestly increased risk of MACE in patients randomly assigned to receive degarelix with wide confidence intervals overlapping the null. The trial was halted due to low enrollment and changes in clinical practice patterns, such as use of chemo-hormonal therapy for patients with metastatic and hormone-sensitive disease (12,28,29).

When excluding death from the primary outcome, the ITT estimates moved closer to the null. In part, this finding may be explained by the fact that death was a driver of the composite outcome in the ITT analysis. Furthermore, there was substantial cross-over in our study, with over 60% of degarelix users switching to leuprolide during follow-up and nearly no treatment switchers in the leuprolide arm (<1%). This pattern was not surprising because there is no current evidence demonstrating a strong advantage of degarelix vs leuprolide in terms of efficacy, and leuprolide offers more convenient dosing and lower injection site reaction rates (30,31). This convenience, coupled with the common treatment pathway of starting with degarelix therapy and subsequently switching to leuprolide to obtain rapid testosterone suppression without a surge, may explain the lower median follow-up we observed among degarelix initiators (32,33).

A major strength of this study is that we designed and registered the study protocol on clinicaltrials.gov before the release of the PRONOUNCE trial results. Thus, our scientific choices were not influenced by previous knowledge of the trial findings. Additionally, the pooled study population is heterogenous, highlighting the advantage of multi-database studies to better understand the reliability of RWE study findings. The rapidly shifting landscape of treatment patterns in oncology also speaks to the need and importance of timely, pragmatic evidence from observational analyses that contrast relevant clinical alternatives.

This study has several limitations. First, although we were able to adjust various potential cardiovascular confounding factors and other markers of health-care use and general health that are well-captured in claims data, residual confounding is a possibility. As observed in our cohort before matching, there were baseline imbalances in risk and health-care–seeking behavior. Most notably, we were not able to directly adjust for several known risk factors for cancer-related mortality—such as tumor stage, histology, and performance status—due to poor documentation in claims alone. Such risk factors may not be confounders for the cardiovascular components of the MACE outcome (eg, myocardial infarction, stroke, and cardiovascular death), but they could be highly relevant confounders for cancer-related death. Assuming a true null effect, we estimated a potential unmeasured confounder would have to have an association with the exposure and outcome of at least 2.0 on the hazard ratio scale to explain the observed association for our primary analysis (34,35).

Second, we included billing codes and pharmacy claims used for different dosing regimens. Consequently, there may be substantial differences between the clinical practice and PRONOUNCE trial treatments. Particularly, dosing frequency may have been lower among leuprolide initiators, which is available in several dosage forms that are administered in longer intervals. Approximately 85% and 25% of leuprolide and degarelix initiators were censored for treatment cessation, respectively, suggesting that total follow-up time of leuprolide initiators may have increased more relative to degarelix users if a longer than 90-day gap was used to determine treatment discontinuation.

Third, death was differentially captured between the data sources. In particular, mortality was well captured in Medicare and Optum claims but incompletely captured in MarketScan (ie, inpatient death only). This differential capture resulted in a greater absolute number of MACE events recorded in Medicare and Optum data relative to MarketScan (Supplementary Table 1, available online). Assuming no other biases, capturing death with a high specificity and relatively low sensitivity, as in MarketScan, is likely to produce unbiased relative effect measures even if absolute rates are inaccurate. This pattern was evident in our on-treatment sensitivity analysis that excluded the death component.

We show a successful emulation of a comparative cardiovascular safety trial in the oncology setting. Although the findings are promising, limitations in measuring cancer stage and progression are challenges in attempting to generalize whether claims-based observational analyses can be used as actionable evidence.

## Funding

This work was supported by the U.S. Food and Drug Administration (grant numbers HHSF223201710186C, HHSF223201710146C).

## Notes

**Role of the funder:** The funders had no role in the design of the study; the collection, analysis, and interpretation of the data; the writing of the manuscript; and the decision to submit the manuscript for publication.

**Author contributions:** Conceptualization: SW, SS Methodology: DM, SW; Data curation and formal analysis: DM, SKS, LZ; Writing—original draft: DM; Writing—review and editing: DM, SW, SKS, LZ, KQ, JC, SS. Project administration: SW, SKS, SS.

**Disclosures:** SS (ORCID# 0000–0003-2575-467X) reports participating in investigator-initiated grants to the Brigham and Women’s Hospital from Boehringer Ingelheim unrelated to the topic of this study. SS is a consultant to Aetion Inc, a software manufacturer of which he owns equity. SS reports that his interests were declared, reviewed, and approved by the Brigham and Women’s Hospital in accordance with their institutional compliance policies. DM reports employment compensation unrelated to the present work and ownership of equity in Aetion, Inc. Authors SW, SKS, LZ, KQ, JC have no conflicts of interest to disclose.

## Supplementary Material

pkac049_Supplementary_Data

## Data Availability

The data underlying this article cannot be shared publicly to protect the privacy of individuals assessed in the study. The data underlying this article were provided by Optum^®^, IBM Watson^®^, and the Centers for Medicare and Medicaid Services under license.
