# Supplementary material for: Real-World Evidence Prediction of a Phase IV Oncology Trial: Comparative Degarelix vs Leuprolide Safety
Source: JNCI Cancer Spectr. 2022 Aug 10;6(4):pkac049. doi: 10.1093/jncics/pkac049 (PMC9403105; doi:10.1093/jncics/pkac049)
Supplement: pkac049_Supplementary_Data [file pkac049_supplementary_data.pdf]

Supplementary Tables

Supplementary Table 1. Primary outcome by database in 1:1 matched cohort

Supplementary Table 2. Primary and secondary outcomes in the intention-to-treat analysis

Supplementary Table 1. Primary outcome by database in 1:1 matched cohort[a]

Major adverse cardiovascular events (MACE)	Degarelix No. of events (IR/1,000 PY)	Leuprolide No. of events (IR/1,000 PY)	HR (95% CI) Ref. = leuprolide	P-value[b]
Clinformatics (n = 1,098)	26 (163.52)	14 (88.05)	1.83 (0.93 - 3.58)	
MarketScan (n = 830)	6 (53.10)	5 (37.59)	1.17 (0.35 - 3.88)	0.567
Medicare - Diabetes (n = 1,850)	41 (163.35)	36 (130.43)	1.19 (0.75 - 1.88)	
Pooled (n = 3,778)	73 (139.58)	55 (96.83)	1.35 (0.94 - 1.93)	-

[a]The availability of mortality information varied by database. Medicare and Optum Clinformatics have more complete mortality data coming from multiple sources, whereas MarketScan only captured death through in-hospital discharge status. CI = confidence interval; HR = hazard ratio; IR = incidence rate; PY = person-years; Ref. = reference

[b]P-value for homogeneity from fixed effects meta-analysis of all three estimates

Supplementary Table 2. Primary and secondary outcomes in the intention-to-treat analysis[a,b]

	Real-World Data			PRONOUNCE Trial		
	Degarelix, (n = 1,889) No. of events (%)	Leuprolide, (n = 1,889) No. of events (%)	HR (95% CI) Ref. = leuprolide	Degarelix, (n = 275) No. of events (%)	Leuprolide, (n = 269) No. of events (%)	HR (95% CI) Ref. = leuprolide
Major adverse cardiovascular event (MACE)	194 (10.3)	138 (7.3)	1.42 (1.14 - 1.76)	15 (5.5)	11 (4.1)	1.32 (0.61 - 2.87)
MACE Components						
All-cause mortality	140 (7.4)	88 (4.7)	1.59 (1.22-2.08)	-	-	-
Acute Myocardial Infarction	58 (3.1)	45 (2.4)	1.30 (0.88-1.92)	-	-	-
Stroke	15 (0.8)	18 (1.0)	0.83 (0.41-1.69)	-	-	-
Composite Myocardial Infarction & Stroke	74 (3.9)	72 (3.8)	1.03 (0.75-1.44)	-	-	-

[a]The availability of mortality information varied by database. Medicare and Optum Clinformatics have more complete mortality data coming from multiple sources, whereas MarketScan only captured death through in-hospital discharge status. CI = confidence interval HR = hazard ratio; Ref. = reference

[b]Median follow-up time to ascertain MACE events in the real-world data study was shorter than that of the PRONOUNCE trial
